# Supplementary material for: PUFA synthase-independent DHA synthesis pathway in Parietichytrium sp. and its modification to produce EPA and n-3DPA
Source: Commun Biol. 2021 Dec 9;4:1378. doi: 10.1038/s42003-021-02857-w (PMC8660808; doi:10.1038/s42003-021-02857-w)
Supplement: Supplementary file 2 — Supplementary information [file 42003_2021_2857_MOESM2_ESM.pdf]

Supplementary Information

PUFA synthase-independent DHA synthesis pathway in *Parietichytrium* sp. and its modification to produce EPA and n-3DPA

Yohei Ishibashi[1#], Hatsumi Goda[1#], Rie Hamaguchi[1#], Keishi Sakaguchi[1#], Takayoshi Sekiguchi[3], Yuko Ishiwata[3], Yuji Okita[3], Seiya Mochinaga[1], Shingo Ikeuchi[1], Takahiro Mizobuchi[1], Yoshitake Takao[4], Kazuki Mori[1], Kosuke Tashiro[1], Nozomu Okino[1], Daiske Honda[5,6], Masahiro Hayashi[7], Makoto Ito[1,2,*]
[1]*Department of Bioscience and Biotechnology,* [2]*Innovative Bio-architecture Center, Faculty of Agriculture, Kyushu University, Fukuoka 819-0395, Japan,* [3] *Central research laboratory, Nippon Suisan Kaisha, Ltd., Tokyo 192-0991, Japan,* [4] *Department of Marine Science and Technology, Faculty of Marine Science and Technology, Fukui Prefecture University, Fukui 917-0003, Japan,* [5]*Department of Biology, Faculty of Science and Engineering,* [6]*Institute for Integrative Neurobiology, Konan University, Hyogo 658-8501, Japan,* [7]*Department of Marine Biology and Environmental Sciences, Faculty of Agriculture, University of Miyazaki, Miyazaki 889-2192, Japan*
#equal contributor

*Corresponding author: Makoto Ito, 744 Motooka, Nishi-ku, Fukuoka 819-0395, Japan
Email: makotoi@agr. kyushu-u.ac.jp

This PDF file includes:
Tables S1
Figures S1 to S13

Supplemental Table 1

Microbial production of EPA and n-3 DPA.

a EPA Production

	EPA (% TFA)	EPA (mg/g-DCW)	EPA (g/L)	References
Phaeodactylum tricornutum	7.8 (Neutral lipid)<br>25.5 (Membrane lipid)	8.3	0.05	(1)
Thalassiosira pseudonana (wild type)<br>*Thalassiosira pseudonana* (Δ6DES OE)	-	19.0	0.001<br>0.554	(2)<br>(3)
Mortierella alpina	30.0<br>26.4	-<br>-	1.3<br>1.8	*WO* 2016/031947<br>(4)
Yarrowia lipolytica	55.6	120.7	5.5	*WO* 2009/046231<br>(5)
Aurantiochytrium sp.	3	-	2.7	(6)
Parietichytrium sp.	15.6	78.9	11.4	Present study

b n-3DPA Production

	n-3DPA (% TFA)	n-3DPA (mg/g-DCW)	n-3DPA (g/L)	References
Phaeodactylum tricornutum	4.5	2.7	-	(7)
Schizochytrium sp.	-	19.6	-	*US* 20160374976
Yarrowia lipolytica	5.7	7.0	0.05	(8)
Parietichytrium sp.	11	57.1	4.8	Present study

References

1. I. M. Remmers, D. E. Martens, R. H. Wijffels, P. P. Lamers, Dynamics of triacylglycerol and EPA production in *Phaeodactylum tricornutum* under nitrogen starvation at different light intensities. *PLoS One* **12**, 1–13 (2017).
2. O. Cook, M. Hildebrand, Enhancing LC-PUFA production in *Thalassiosira pseudonana* by overexpressing the endogenous fatty acid elongase genes. *J. Appl. Phycol.* **28**, 897–905 (2016).
3. H. Shi, X. Luo, R. Wu, X. Yue, Production of eicosapentaenoic acid by application of a delta-6 desaturase with the highest ALA catalytic activity in algae. *Microb. Cell Fact.* **17**, 7 (2018).
4. T. Okuda, *et al.*, Eicosapentaenoic acid (EPA) production by an oleaginous fungus *Mortierella alpina* expressing heterologous the Δ17-desaturase gene under ordinary temperature. *Eur. J. Lipid Sci. Technol.* **117**, 1919–1927 (2015).
5. Z. Xue, *et al.*, Production of omega-3 eicosapentaenoic acid by metabolic engineering of *Yarrowia lipolytica*. *Nat. Biotechnol.* **31**, 734–740 (2013).
6. S. Wang, *et al.*, Optimizing Eicosapentaenoic Acid Production by Grafting a Heterologous Polyketide Synthase Pathway in the Thraustochytrid *Aurantiochytrium*. *J. Agric. Food Chem.* **68**, 11253–11260 (2020).
7. M. L. Hamilton, *et al.*, Towards the industrial production of omega-3 long chain polyunsaturated fatty acids from a genetically modified diatom *Phaeodactylum tricornutum*. *PLoS One* **10**, e0144054 (2015).
8. K. Gemperlein, *et al.*, Polyunsaturated fatty acid production by *Yarrowia lipolytica* employing designed myxobacterial PUFA synthases. *Nat. Commun.* **10**, 4055 (2019).

a

Elongase (ELO)

Substrate→Product	Mock	ELO-1	ELO-2	ELO-3
PA →SA	26.8	**55.9**	29.7	29.7
GLA →DGLA	0.5	0.0	**77.2**	3.3
LA→EDA	0.0	0.0	24.5	1.4
ARA→DTA	2.2	2.8	11.1	**43.9**
STA→ETA	1.5	1.4	**77.0**	8.4
ALA→ETrA	0.5	2.3	33.3	1.5
EPA→n-3DPA	0.8	1.0	15.7	**80.4**
Putative function		**C16ELO**	**C18ELO > C20ELO**	**C20ELO**

Desaturase (DES)

Substrate→Product	Mock	DES-1	DES-2	DES-3	DES-4	DES-5
SA→OA	80.8	**97.1**	69.3	74.6	76.3	76.0
OA→LA	17.3	11.2	**49.2**	11.1	25.9	25.0
LA→GLA	0.0	0.0	0.3	**68.5**	0.0	0.0
EDA→DGLA	0.0	0.0	0.0	**42.5**	3.4	0.0
DGLA→ARA	0.0	0.0	0.0	3.1	**69.2**	3.9
DTA→n-6DPA	0.0	0.0	0.0	0.0	0.0	**18.8**
ALA→STA	1.0	0.4	0.9	**71.8**	0.6	0.7
ETrA→ETA	1.6	0.0	1.3	**57.5**	0.0	0.0
ETA→EPA	0.0	3.0	0.0	1.2	**66.2**	1.2
n-3DPA→DHA	3.4	0.0	0.0	1.4	0.0	**11.0**
Putative function		**Δ9DES**	**Δ12DES**	**Δ6DES > Δ8DES**	**Δ5DES**	**Δ4DES**

Substrate→Product	Mock	DES-6
LA→ALA	0.0	0.3
GLA→STA	0.1	0.4
EDA→EtrA	0.1	0.4
DGLA→ETA	0.4	**1.2**
ARA→EPA	1.5	**6.2**
DTA→n-3DPA	0.0	**1.9**
n-6DPA→DHA	0.7	**4.4**
Putative function		**ω3DES (Δ17, Δ19)**

b

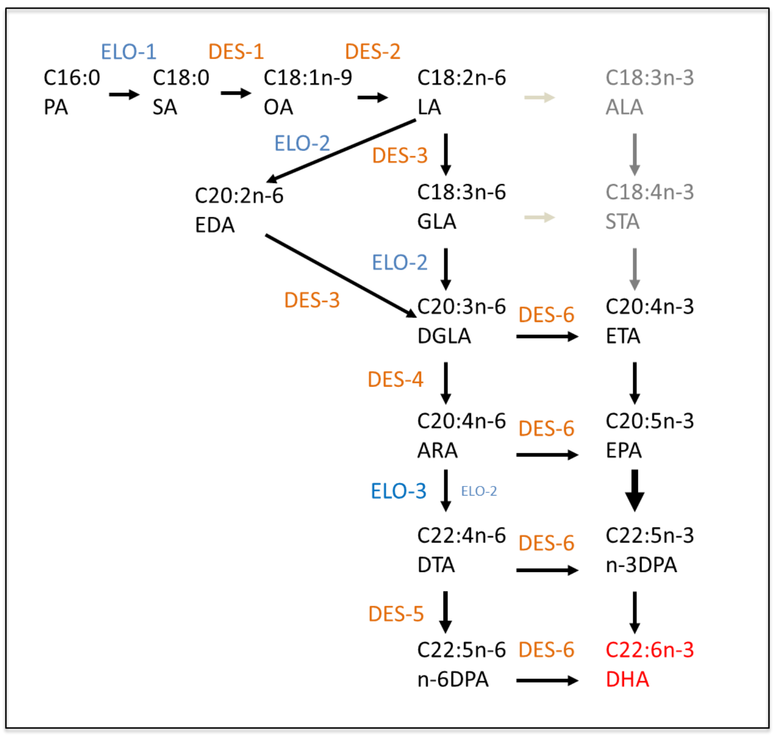

c

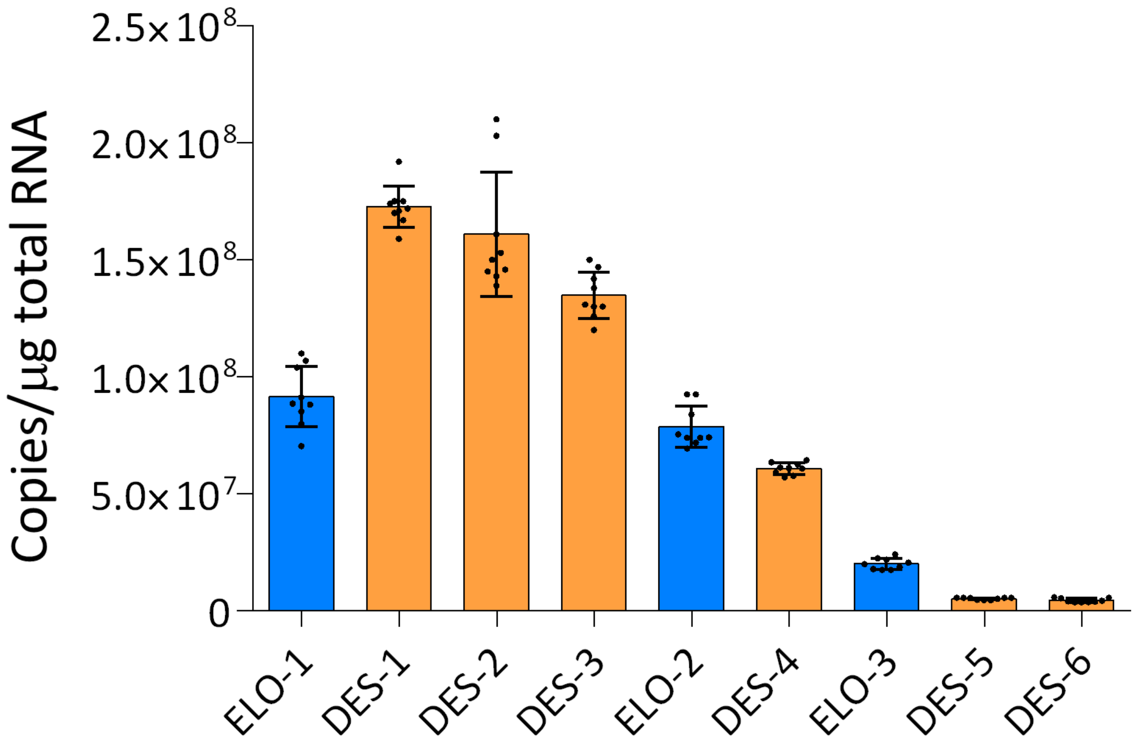

Supplemental Fig. S1. Identification and characterization of ELOs and DESs involved in the DHA synthesis pathway in *Parietichytrium* sp.

a Activities of ELOs and DESs of *Parietichytrium* sp. for different fatty acid substrates. Each gene was expressed and analyzed using the *S. cerevisiae* expression system, as described in Methods. Fatty acids of *S. cerevisiae* were analyzed by GC, and activity was calculated as follows: Activity (%) = GC area for product fatty acid x 100/(GC area for substrate fatty acid + GC area for product fatty acid). Marked activity is highlighted by the colored box. **b** Schematic diagram showing 3 ELOs and 6 DESs involved in the DHA synthesis pathway. **c** mRNA expression levels of 3 ELOs and 6 DESs in *Parietichytrium* sp. SEK358 was cultured in 20 mL of GY medium at 25°C for 2 days with shaking at 120 rpm. Quantitative real-time PCR was performed as described in Methods. The data shown are the mean ± S.D. (n=3). The n values are numbers of replicates.

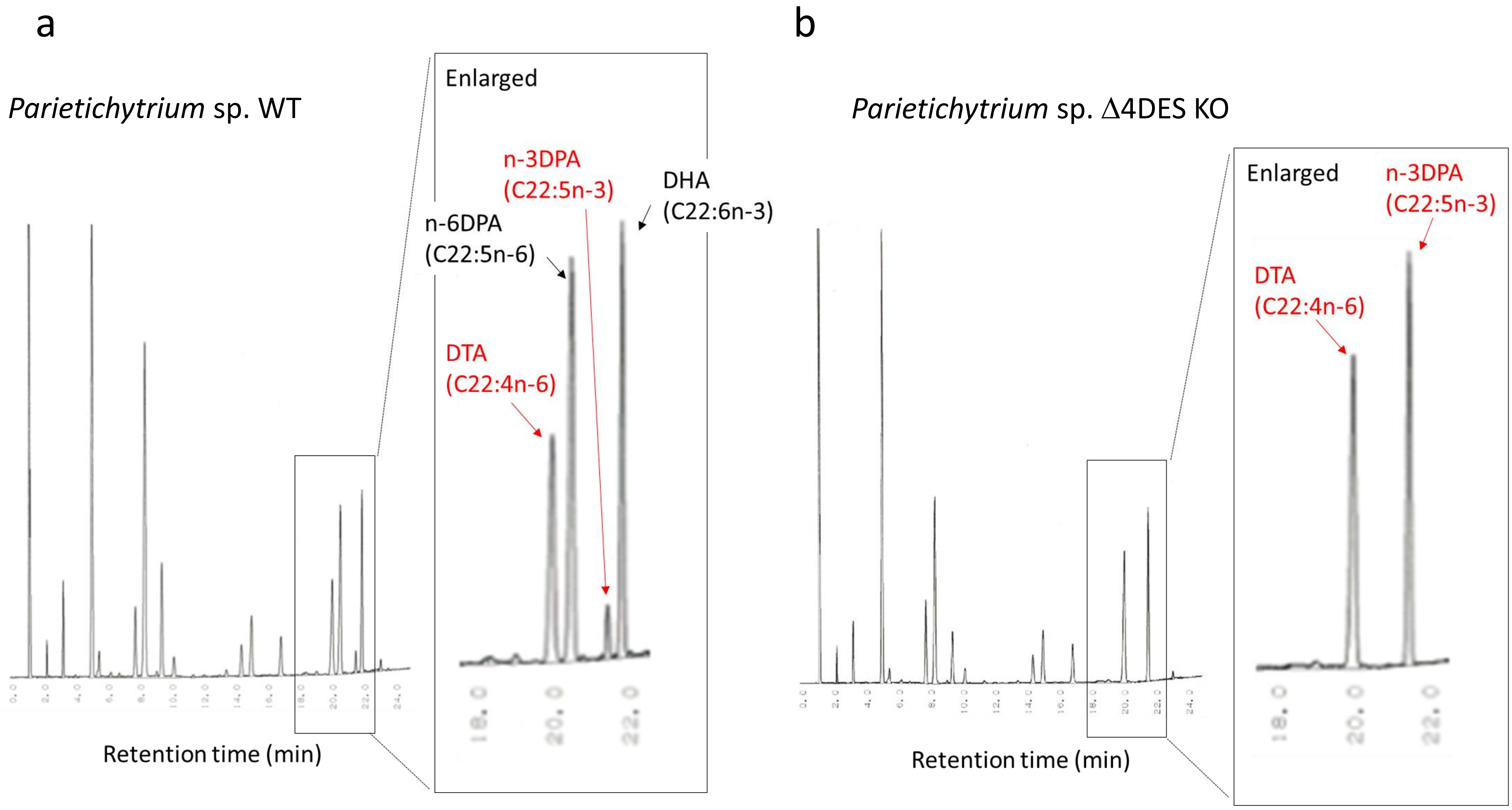

Supplemental Fig. S2. Gas chromatography (GC) showing the effects of Δ4DES gene disruption in *Parietichytrium* sp.

GC charts of WT **a** and Δ4 DES KO **b** strains of *Parietichytrium* sp. SEK358. Fatty acids were measured as FAMEs using GC as described in Methods.

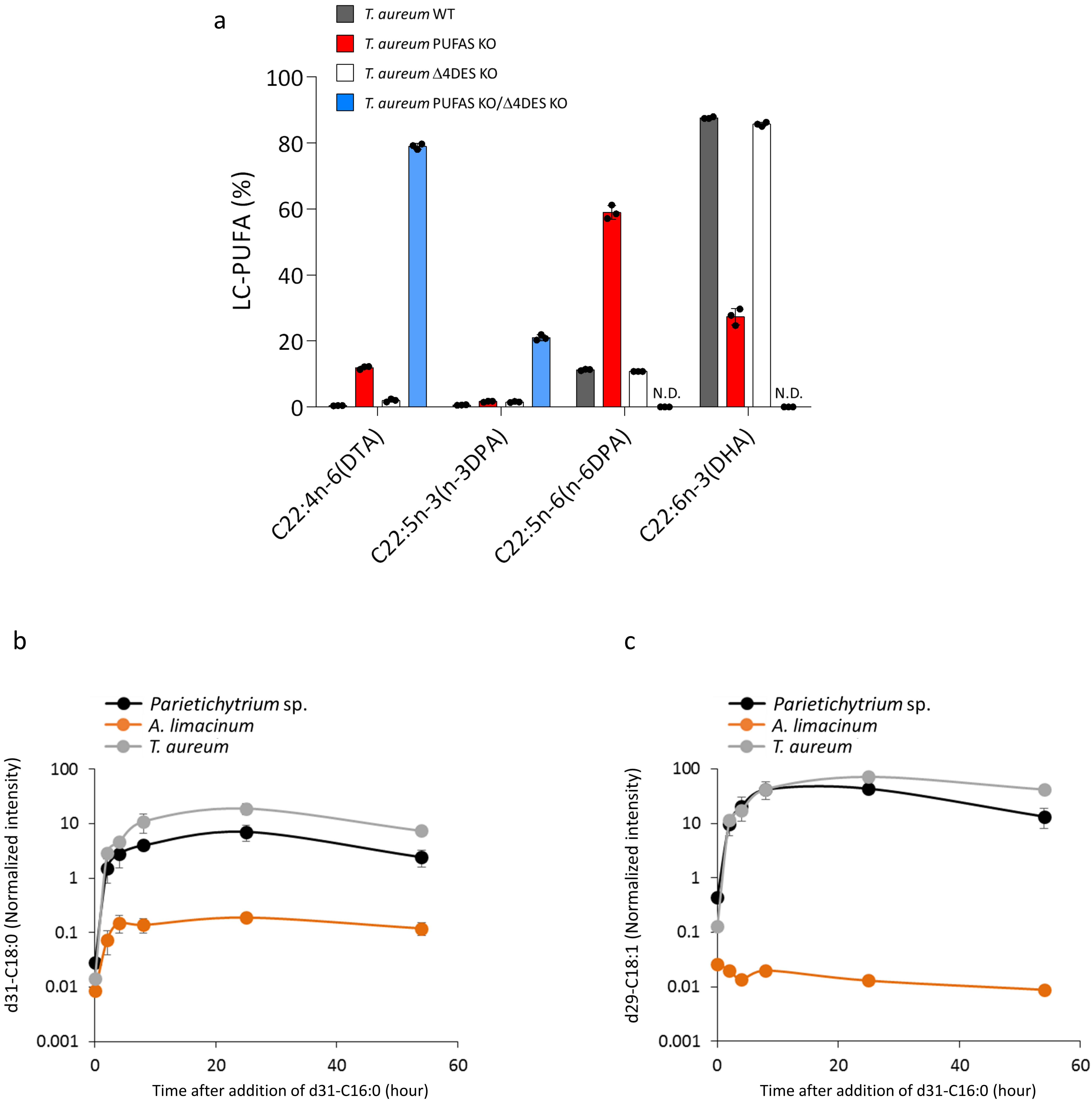

Supplemental Fig. S3. Contribution of ELO/DES pathway to DHA synthesis in *T. aureum* and *A. limacinum.*

a Effects of disruption of Δ4DES and PUFA-S on DHA synthesis in *T. aureum*. N.D., not detected. The data shown are the mean ± S.D. (n=3). Time-course of the generation of deuterium-labeled C18:0 (d31-C18:0) **b** and C18:1 (d29-C18:1) **c** in three different genera of thraustochytrids. Cells were incubated with 0.5 mM d31-C16:0 and collected at the indicated time points. The d31-18:0 and d29-18:1 were obtained from the total lipid fractions and determined by LC-ESI MS/MS analysis. The data shown are the mean ± S.D. (n=3). The n values are numbers of replicates. The vertical axis is represented as a logarithmic scale.

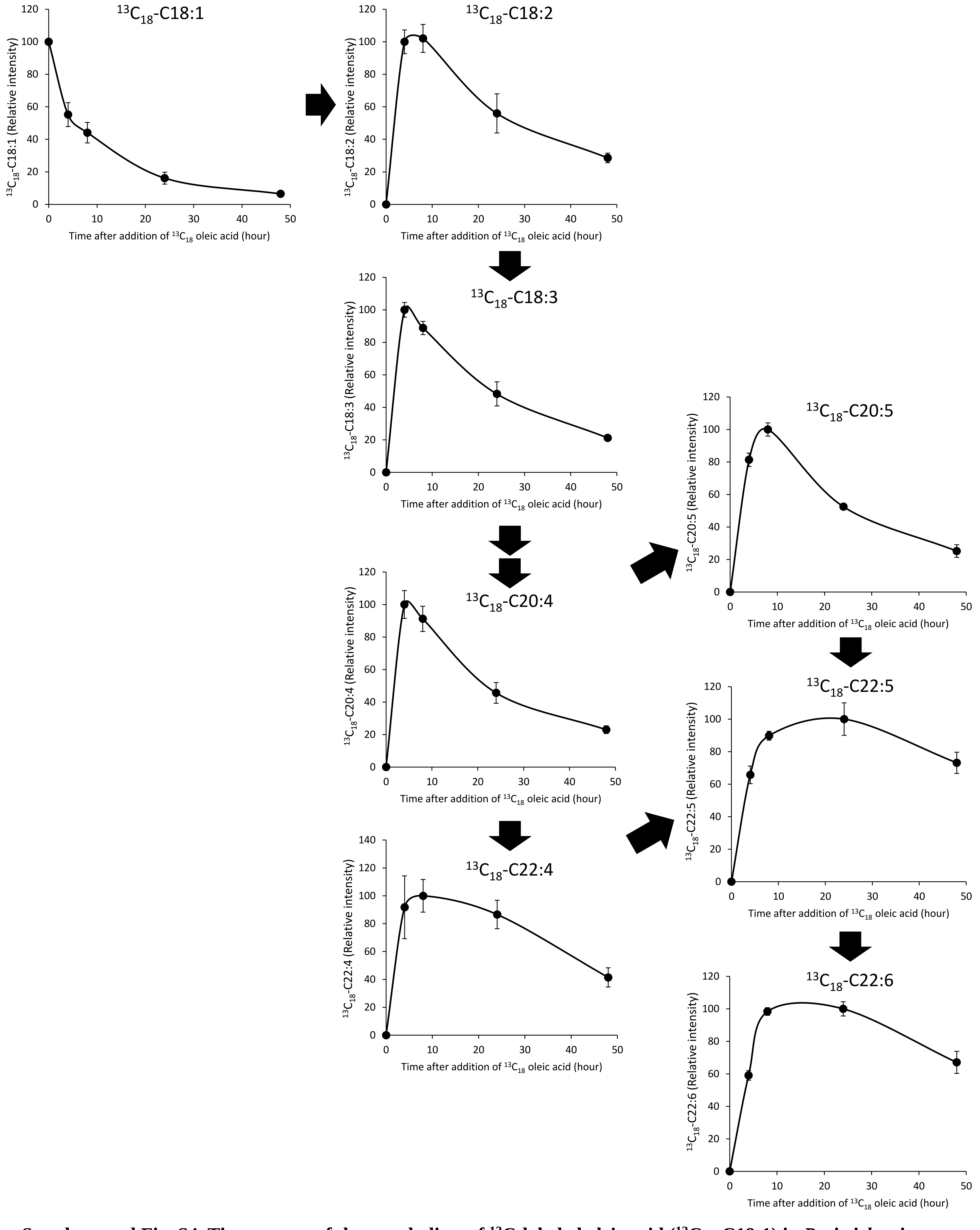

Supplemental Fig. S4. Time-course of the metabolism of ^{13}C-labeled oleic acid ($^{13}C_{18}$-C18:1) in *Parietichytrium* sp. by LC-ESI MS/MS.

Parietichytrium sp. SEK358 was incubated with 0.25 mM $^{13}C_{18}$-C18:1 and collected at the indicated time-points. The ^{13}C-labeld fatty acids were measured by LC-ESI MS/MS analysis. The data shown are the mean ± S.D. (n=3). The n values are numbers of replicates.

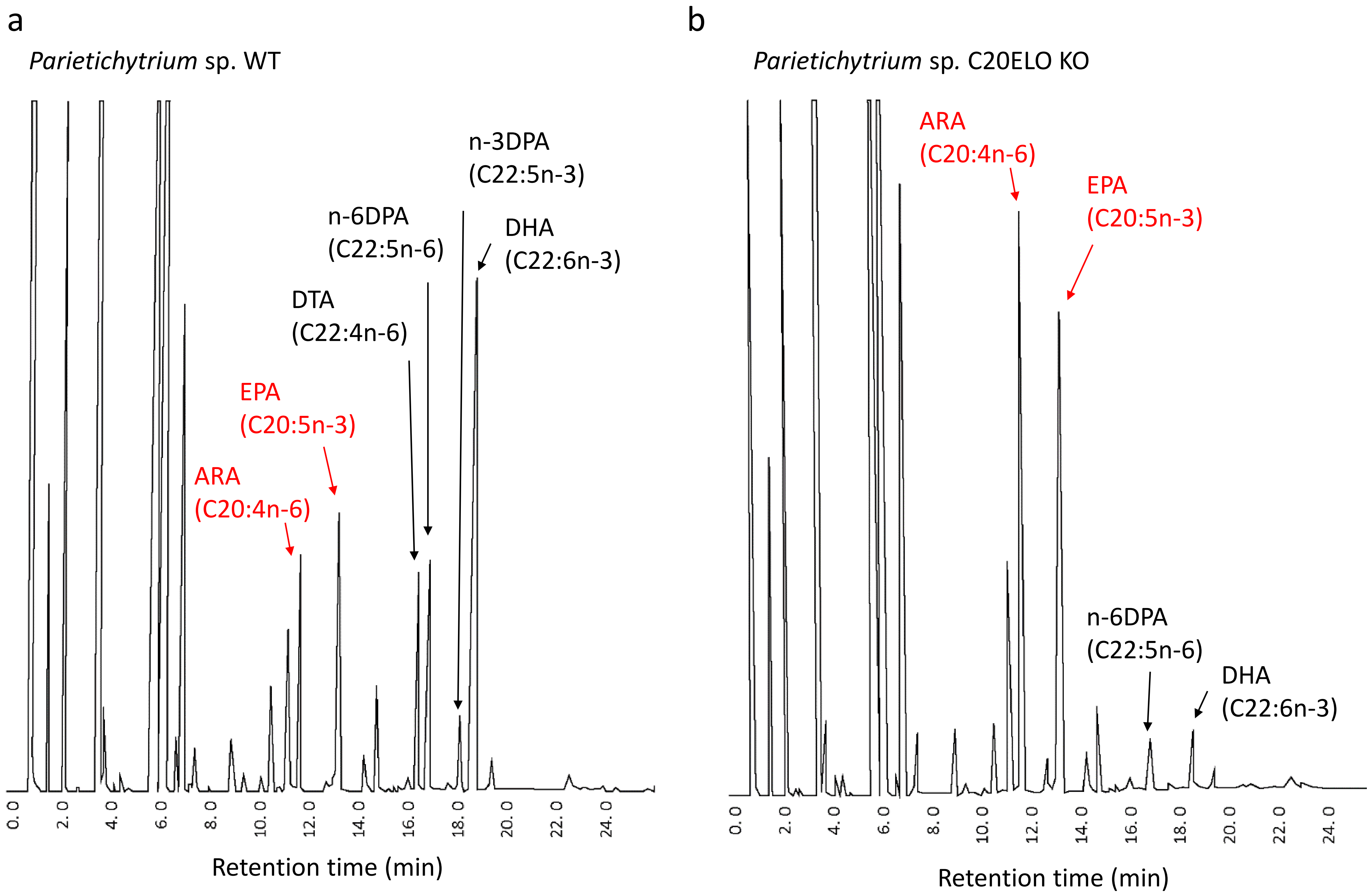

Supplemental Fig. S5. GC showing the effects of C20ELO gene disruption in *Parietichytrium* sp.

GC charts of WT **a** and C20ELO KO **b** strains of *Parietichytrium* sp. SEK358. Fatty acids were measured as FAMEs using GC as described in Methods.

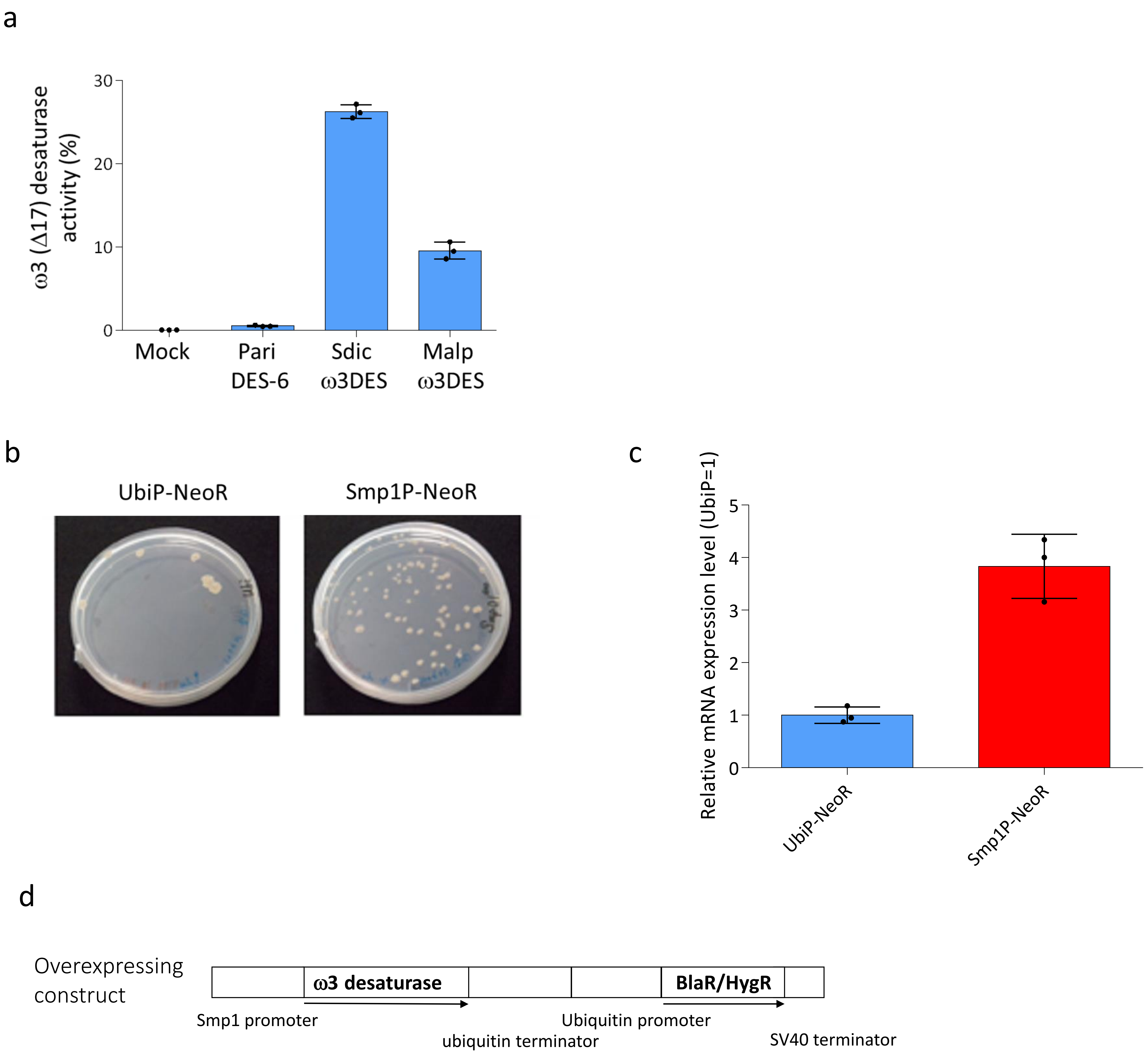

Supplemental Fig. S6. Selection of ω3DES and its expression in *Parietichytrium* sp. using a virus-derived high-expression promoter.

a ω3DES activity of DES-6 (*Parietichytrium* sp.), Sdic ω3DES (*S. diclina*), and Malp ω3DES (*M. alpina*). DES activity was measured by heterologous expression of each DES in *S. cerevisiae*. ARA (C20:4n-6) was added to the culture of *S. cerevisiae* harboring each DES gene, and the conversion of ARA to EPA was measured by GC analysis. Activity (%) = GC area of EPA x 100/(GC area of ARA + GC area of EPA). **b** Transformation of *Parietichytrium* sp. by NeoR driven by the *T. aureum* ubiquitin promoter (UbiP) and SmDNAV-derived promoter (Smp1P). *Parietichytrium* sp. was transformed with a construct containing NeoR driven by each promoter and plated on a YPD agar plate containing G418. **c** Relative mRNA expression level of NeoR driven by Ubi and Smp1 promoters. The data shown are the mean ± S.D. (n=3). The n values are numbers of replicates. **d** Schematic diagram of a DNA construct for the overexpression of *S. diclina* ω3DES in *Parietichytrium* sp. A BlaR-containing construct was used to establish C20ELO KO/ω3DES OE in *Parietichytrium* sp. SEK358 and SEK364. A HygR-containing construct was used to establish Δ4DES KO/ω3DES OE in *Parietichytrium* sp. SEK358.

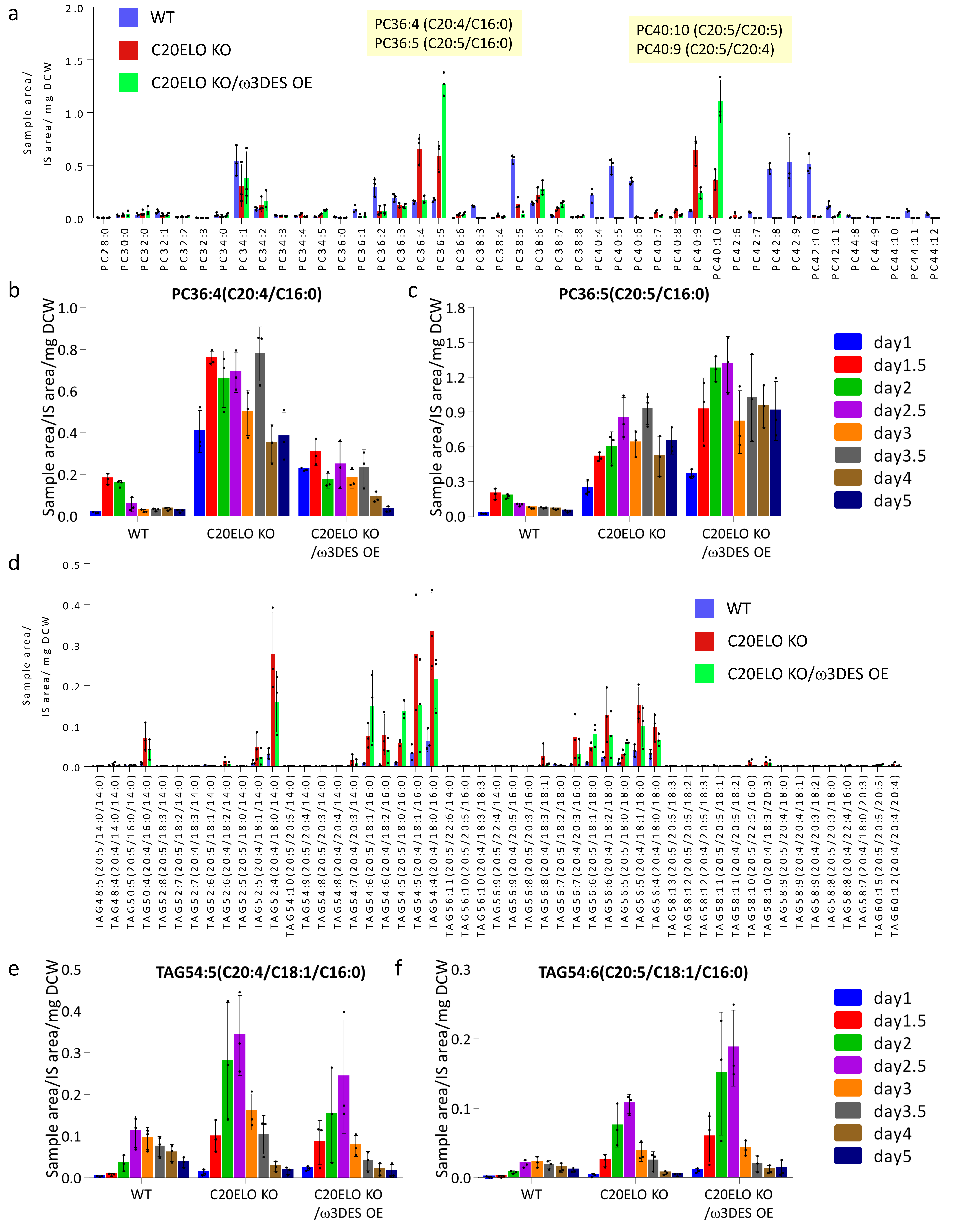

Supplemental Fig. S7

Supplemental Fig. S7. Analyses of PC and TAG possessing ARA or EPA in *Parietichytrium* sp. wildtype (WT) and mutant strains by LC-ESI MS/MS.

Quantification of phospholipids (PC) and neutral lipids (TAG) that possess C20:4 (ARA) or C20:5 (EPA) of WT and mutant strains of *Parietichytrium* sp. SEK358. **a** Composition of PC molecules in WT, C20ELO KO, and C20ELO KO/ω3DES OE strains cultured for 2 days. PC molecules possessing C20:4 or C20:5 are shown in the yellow box. Time-course of the production of PC36:4 and PC36:5 in WT and mutant strains of *Parietichytrium* sp. SEK358. is shown in **b** and **c**, respectively. **d** Composition of TAG molecules possessing C20:4 or C20:5 in WT, C20ELO KO, and C20ELO KO/ω3DES OE strains cultured for 3 days. Time-course of the production of TAG54:5 and TAG54:6 is shown in **e** and **f**, respectively. Each PC and TAG molecule was measured by MRM analysis using LC-ESI MS/MS, as shown in the legend of Supplementary data set. The peak intensity of each molecule was normalized by the internal standard and DCW. All data shown are based on the normalized intensities and are the mean ± S.D. (n=3). The n values are numbers of replicates. The peak intensity of PC on LC-ESI MS/MS is much higher than that of TAG, as shown in Supplemental Fig. S9, and, thus, it is impossible to compare the amounts of phospholipids with those of TAGs in these figures.

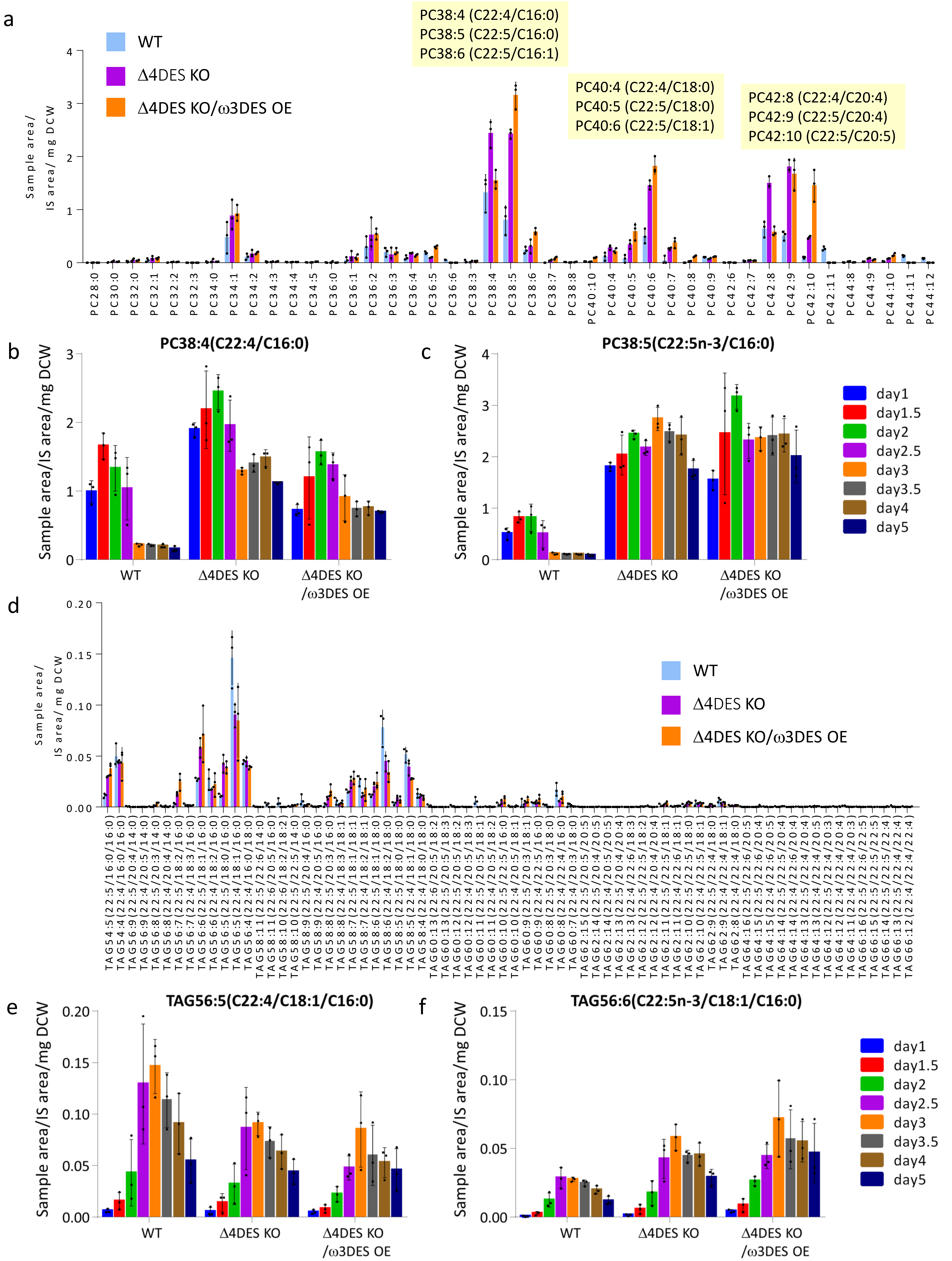

Supplemental Fig. S8

Supplemental Fig. S8. Analysis of PC and TAG possessing DTA or n-3DPA in *Parietichytrium* sp. WT *and* mutant strains by LC-ESI MS/MS.

Quantification of phospholipids (PCs) and neutral lipids (TAGs) that possess C22:4 (DTA) and C22:5n-3 (n-3DPA) in WT and mutant strains of *Parietichytrium* sp. SEK358. **a** Composition of PC in WT, Δ4DES KO, and Δ4DES KO/ω3DES OE strains cultured for 2 days. PC possessing C22:4 or C22:5n-3 is shown in the yellow box. The *m/z* of C22:5n-3 is the same as that of C22:5n-6, but PC possessing C22:5n-3 was distinguished from that possessing C22:5n-6 due to the difference in retention time on LC. Time-course of the production of PC38:4 and PC38:5 in *Parietichytrium* sp. SEK358 and its mutant strains is shown in **b** and **c**, respectively. **d** Composition of TAGs possessing C22:4 or C22:5n-3 in WT, Δ4DES KO, and Δ4DES KO/ω3DES OE strains cultured for 2.5 days. Time-course of the production of TAG56:5 and TAG56:6 in *Parietichytrium* sp. SEK358 and its mutant strains is shown in **e** and **f**, respectively. PCs and TAGs were measured by MRM analysis using LC-ESI MS/MS, as shown in Supplementary data set. The peak intensity of each molecule was normalized by an internal standard and DCW. All data shown are the mean ± S.D. (n=3). The n values are numbers of replicates. Peak intensity of PC on LC-ESI MS/MS is much higher than that of TAG as shown in Supplemental Fig. S9, and, thus, it is impossible to compare the amounts of phospholipids with those of TAGs in these figures.

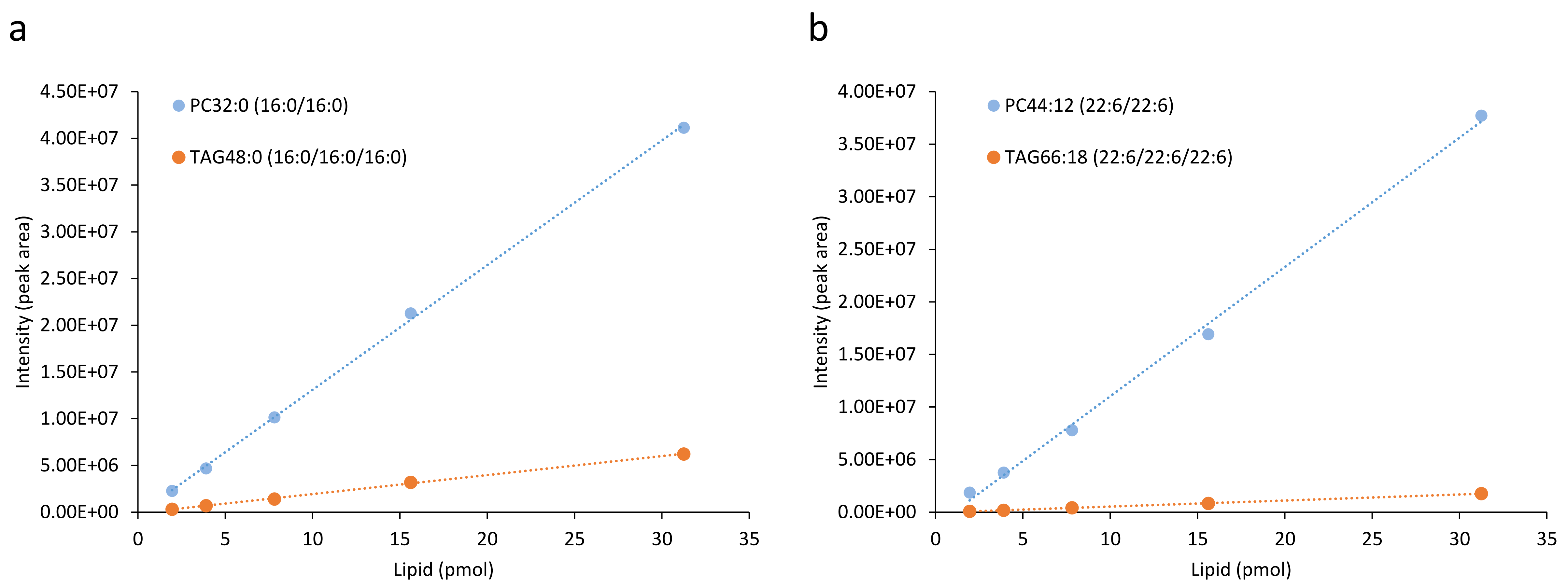

Supplemental Fig. S9. Comparison of peak intensities of PC and TAG on LC-ESI MS/MS.

Relationship between peak intensities and concentrations of PC and TAG possessing C16:0 **a** and C22:6 **b** on LC-ESI MS/MS. Both panels show that the intensity of PC is much higher than that of TAG when measured by MRM analysis using LC-ESI MS/MS.

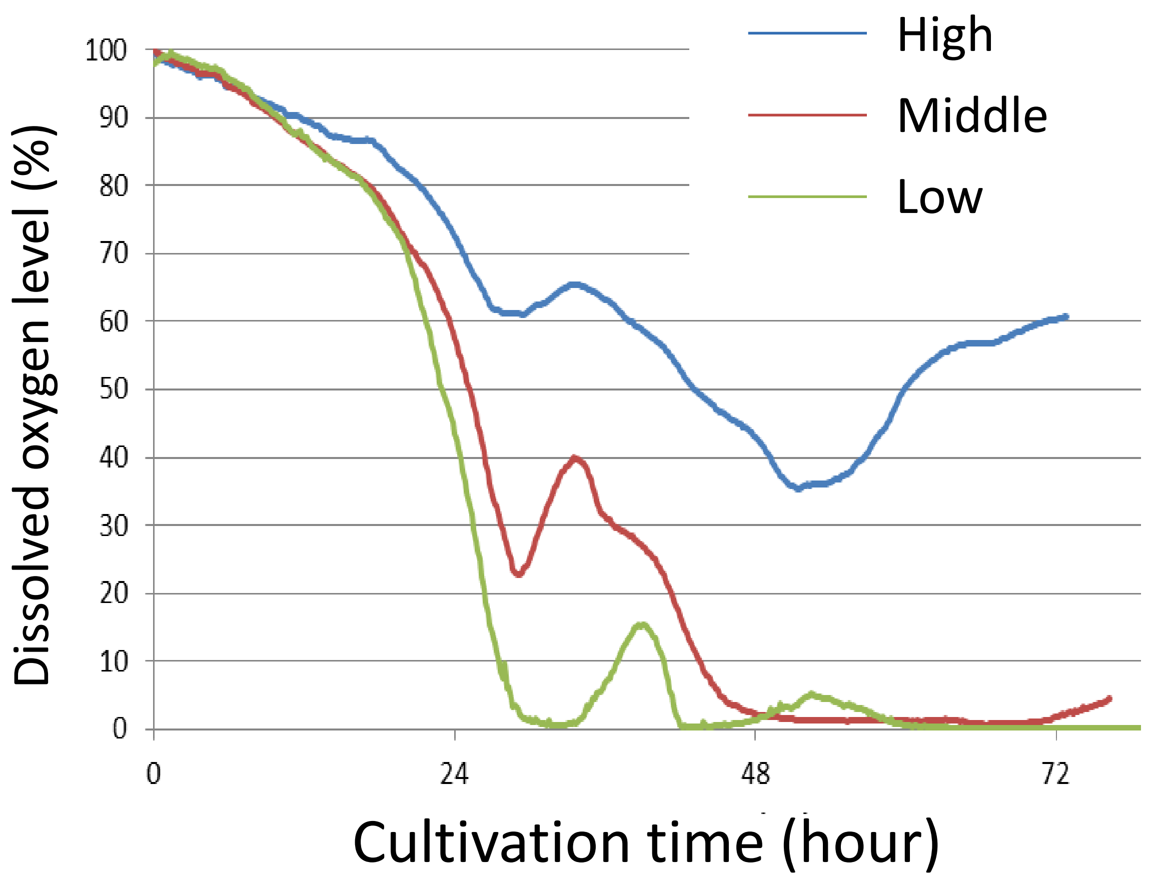

Supplemental Fig. S10. Dissolved oxygen (DO) levels in flask cultures of *Parietichytrium* sp.

DO levels of 100 mL (high DO), 200 mL (middle DO), and 300 mL (low DO) of GY medium in a 500-mL flask with shaking at 25°C were monitored by a DO meter (Galvanic electrochemical DO sensor SDOCφ12 mm, Biott Co., Ltd., Japan).

a

	Culture conditions			
Medium composition	1st	2nd	3rd	4th
Glucose (g/L)	30	60	30	30
Yeast Extract (g/L)	10	20	20	20
Artificial sea water (ml/L)	500	500	500	500
Vitamin mixture solution	1	1	1	1
Element elements solution	1	1	1	1
Feed solution	1st	2nd	3rd	4th
Glucose (g/L)	500	500	500	500
Monosodium Glutamate (g/L)	0	0	38.5	31.7
Ammonium Sulfate (g/L)	0	0	15	11.2
KH_2PO_4 (g/L)	0	0	6	2
K_2HPO_4 (g/L)	0	0	6	11.2

b c

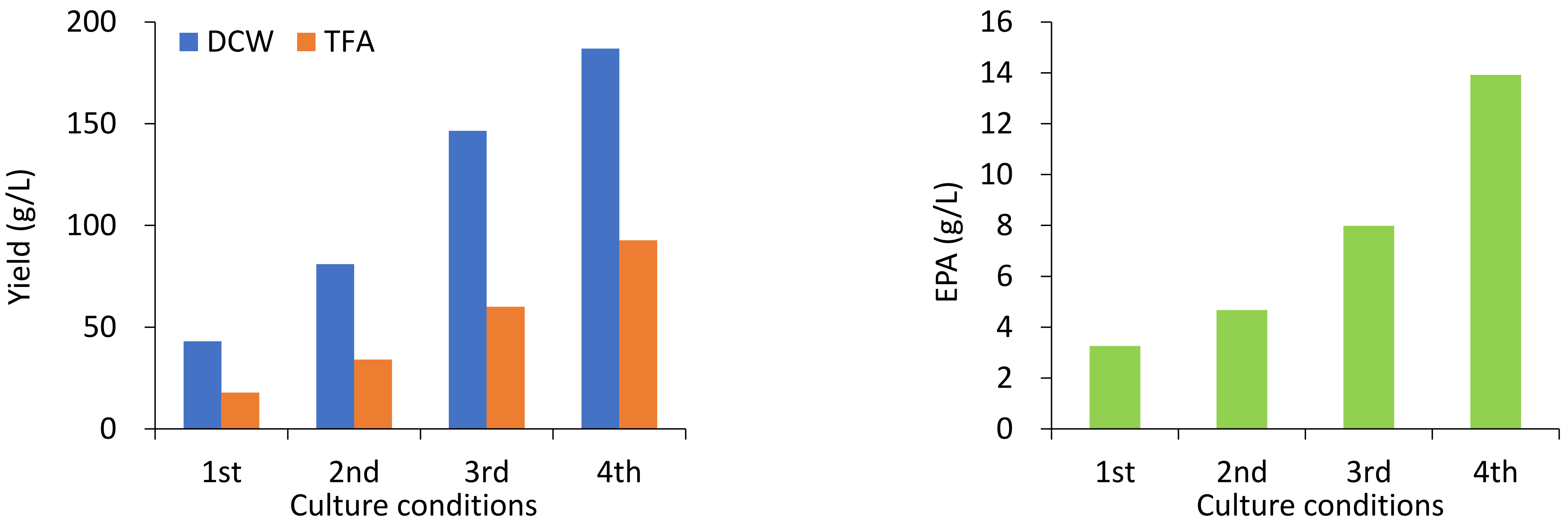

Supplemental Fig. S11. Optimization of fed-batch culture for production of EPA by C20ELO KO/ω3DES OE strain.

a Culture conditions showing the concentration of each component in the medium and feed. **b** The yields of DCW and TFA of C20ELO KO/ω3DES OE under the four different culture conditions. **c** The yield of EPA (g/L) of C20ELO KO/ω3DES OE under the four different culture conditions. Fed-batch culture was conducted using a mutant strain derived from *Parietichytrium* sp. SEK364 under the four different conditions described in (A) at 26°C with aeration (1 vvm) in a 1-liter jar fermenter. Each culture was continued until glucose was depleted. The compositions of vitamin mixture and elements solution are shown in Materials and Methods. ‘vvm’ indicates volume (gas) per volume (medium) per minute.

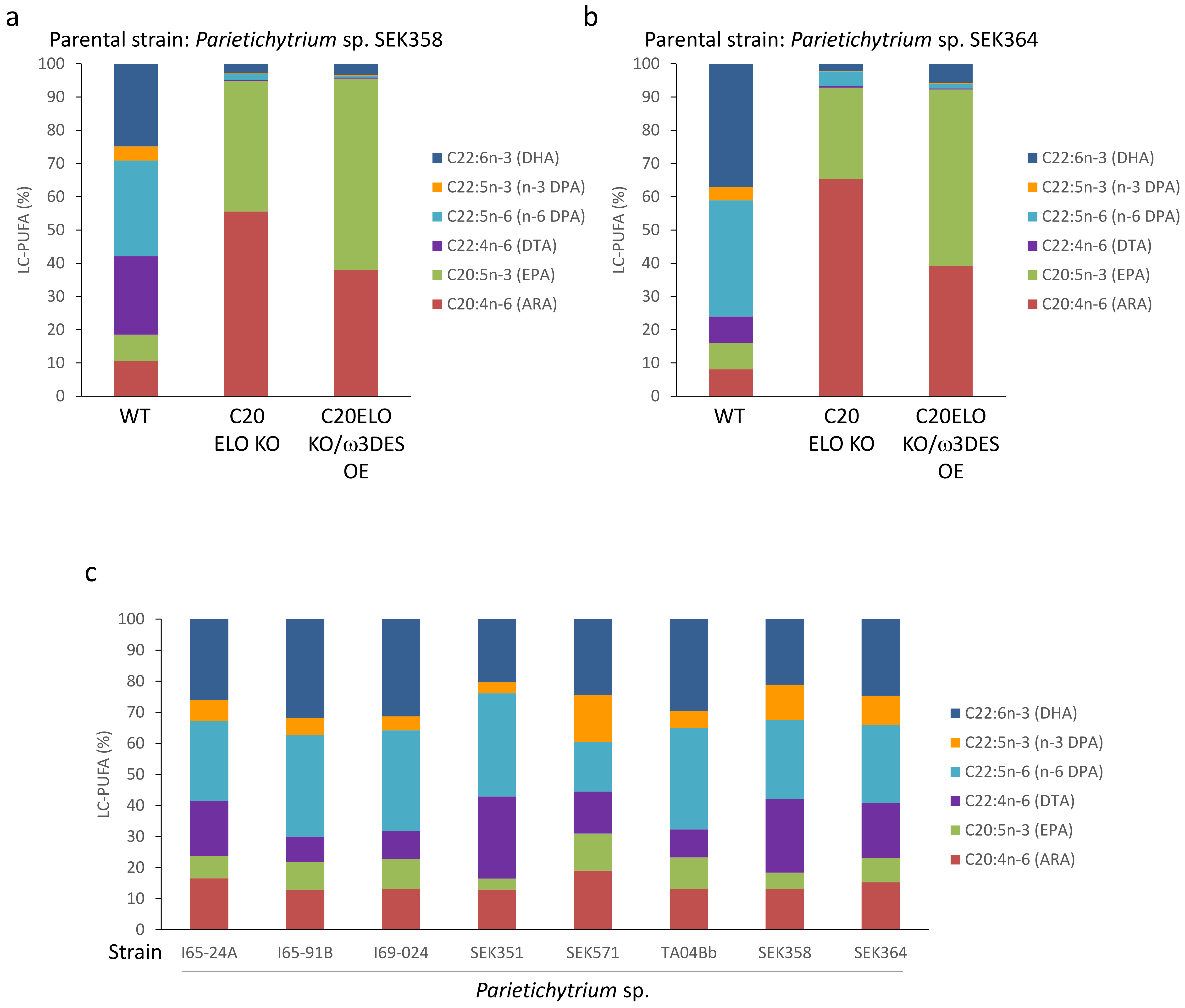

Supplemental Fig. S12. Comparison of LC-PUFA profiles of WT and mutant strains of *Parietichytrium* sp. SEK358 and SEK364, and various WT strains of *Parietichytrium* sp..

C20ELO KO and C20ELO KO/ω3DES OE mutants were generated from *Parietichytrium* sp. SEK358 **a** and SEK364 **b** as a parent strain, respectively, as described in Methods. WT and mutant strains were cultured in a 100-mL flask containing 40 mL of GY medium with 0.1% vitamin mixture and 0.2% trace elements at 25°C for 3 days. Fatty acids were extracted from harvested cells and analyzed by GC, as described in Methods. **c** 8 strains of *Parietichytrium* sp. were cultured in a 100-mL flask containing 20 mL of GY medium with 0.1% vitamin mixture and 0.2% trace elements at 28°C for 3 days.

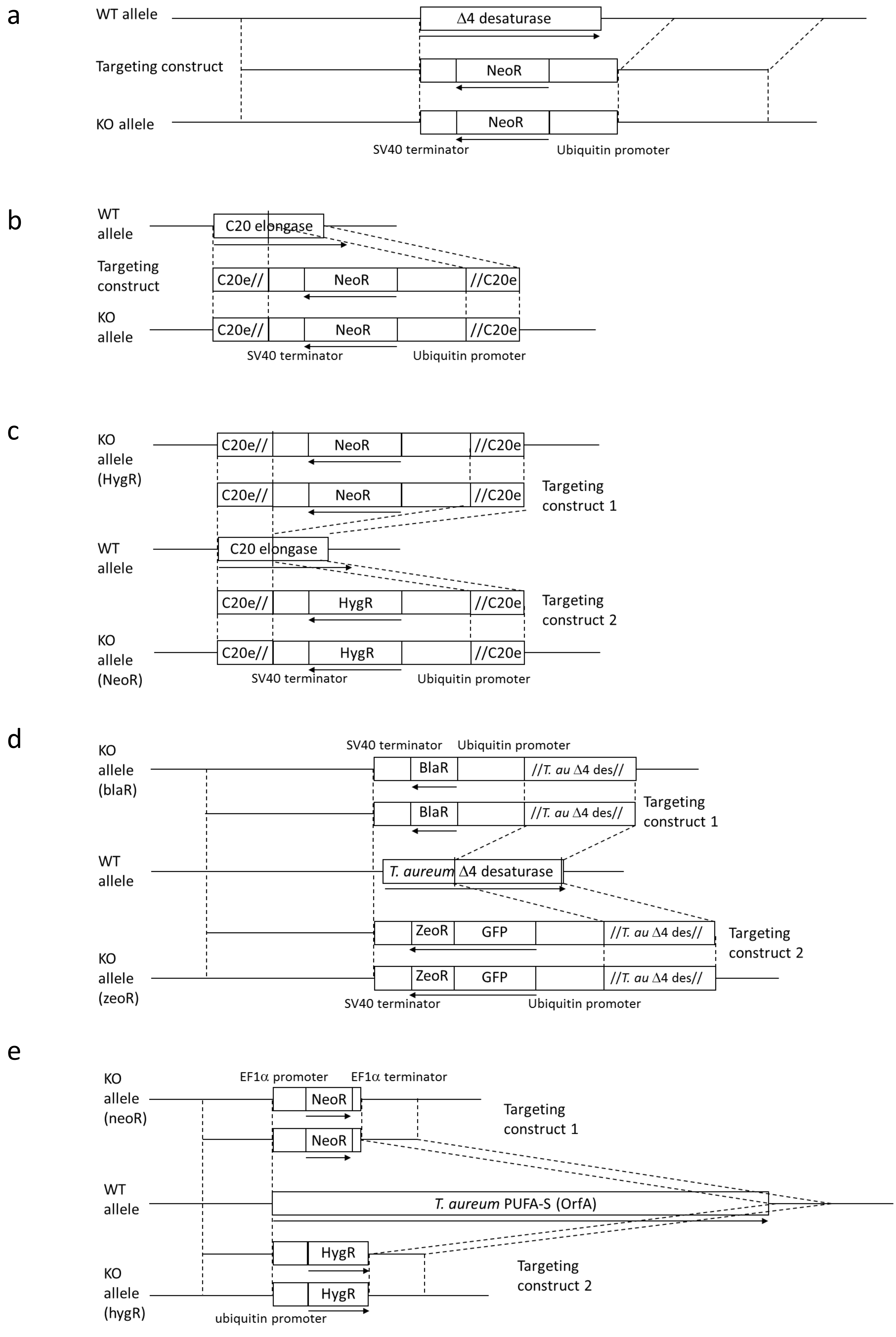

Supplemental Fig. S13. Knockout strategy for Δ4DES, C20ELO, and PUFA-S in *Parietichytrium* sp. and *T. aureum.*

Schematic diagrams of DNA constructs for the disruption of **a** Δ4DES and **b** C20ELO in *Parietichytrium* sp. SEK358, **c** C20ELO in *Parietichytrium* sp. SEK364, and **d** Δ4DES and **e** PUFA-S in *T. aureum* by homologous recombination.
