# Supplementary material for: PUFA synthase-independent DHA synthesis pathway in Parietichytrium sp. and its modification to produce EPA and n-3DPA
Source: Commun Biol. 2021 Dec 9;4:1378. doi: 10.1038/s42003-021-02857-w (PMC8660808; doi:10.1038/s42003-021-02857-w)
Supplement: Supplementary file 3 — Description of Additional Supplementary Files [file 42003_2021_2857_MOESM3_ESM.pdf]

Description of Additional Supplementary Files

File name: Supplementary Data 1.
Description: MRM conditions for the detection of non-labeled fatty acid, 13C-, and deuterium-labeled fatty acid by LC-ESI MS/MS.

File name: Supplementary Data 2.
Description:
Fig. S1. Identification and characterization of ELOs and DESs involved in the DHA synthesis pathway in *Parietichytrium* sp.

Fig. S3. Contribution of ELO/DES pathway to DHA synthesis in *T. aureum* and *A. limacinum*.

Fig. S4. Time-course of the metabolism of ^{13}C-labeled oleic acid (^{13}C18-C18:1) in *Parietichytrium* sp. by LC-ESI MS/MS.

Fig. S6. Selection of •3DES and its expression in *Parietichytrium* sp. using a virus-derived high-expression promoter.

Fig. S7. Analyses of PC and TAG possessing ARA or EPA in *Parietichytrium* sp., wildtype (WT) and mutant strains by LC-ESI MS/MS.

Fig. S8. Analysis of PC and TAG possessing DTA or n-3DPA in *Parietichytrium* sp. mutant strains by LC-ESI MS/MS.

Fig. S9. Comparison of peak intensities of PC and TAG on LC-ESI MS/MS.

Fig. S10. Dissolved oxygen (DO) levels in flask cultures of *Parietichytrium* sp.

Fig. S11. Optimization of fed-batch culture for production of EPA by C20ELO KO/•3DES OE strain.

Fig. S12. Comparison of LC-PUFA profiles of WT and mutant strains of *Parietichytrium* sp. SEK358 and SEK364, and various WT strains of *Parietichytrium* sp.

Fig. S13. Knockout strategy for Δ4DES, C20ELO, and PUFA-S in *Parietichytrium* sp. and *T. aureum*.
