# Supplementary material for: PUFA synthase-independent DHA synthesis pathway in Parietichytrium sp. and its modification to produce EPA and n-3DPA
Source: Commun Biol. 2021 Dec 9;4:1378. doi: 10.1038/s42003-021-02857-w (PMC8660808; doi:10.1038/s42003-021-02857-w)
Supplement: Supplementary file 6 — Reporting Summary [file 42003_2021_2857_MOESM6_ESM.pdf]

nature research

Corresponding author(s): Makoto Ito

Last updated by author(s): September 30, 2021

Reporting Summary

Nature Research wishes to improve the reproducibility of the work that we publish. This form provides structure for consistency and transparency in reporting. For further information on Nature Research policies, see Authors & Referees and the Editorial Policy Checklist .

Statistics

For all statistical analyses, confirm that the following items are present in the figure legend, table legend, main text, or Methods section.

n/a	Confirmed	
☐	☒	The exact sample size (*n*) for each experimental group/condition, given as a discrete number and unit of measurement
☒	☐	A statement on whether measurements were taken from distinct samples or whether the same sample was measured repeatedly
☒	☐	The statistical test(s) used AND whether they are one- or two-sided<br>*Only common tests should be described solely by name; describe more complex techniques in the Methods section.*
☒	☐	A description of all covariates tested
☒	☐	A description of any assumptions or corrections, such as tests of normality and adjustment for multiple comparisons
☐	☒	A full description of the statistical parameters including central tendency (e.g. means) or other basic estimates (e.g. regression coefficient) AND variation (e.g. standard deviation) or associated estimates of uncertainty (e.g. confidence intervals)
☒	☐	For null hypothesis testing, the test statistic (e.g. *F*, *t*, *r*) with confidence intervals, effect sizes, degrees of freedom and *P* value noted<br>*Give P values as exact values whenever suitable.*
☒	☐	For Bayesian analysis, information on the choice of priors and Markov chain Monte Carlo settings
☒	☐	For hierarchical and complex designs, identification of the appropriate level for tests and full reporting of outcomes
☒	☐	Estimates of effect sizes (e.g. Cohen's *d*, Pearson's *r*), indicating how they were calculated

Our web collection on statistics for biologists contains articles on many of the points above.

Software and code

Policy information about availability of computer code

Data collection	The draft genome sequences were obtained using a 454 GS FLX system, and assembled using Newbler assembler version 2.7 software.<br>BioEdit ver. 7.2.0 was used for the local BLAST analysis.<br>The peak intensity of LC ESI MS/MS was analyzed using MultiQuant 3.0.1.
Data analysis	MS Excel and Graphpad Prism 6.0 softwarefor for bar charts and line graphs<br>MS Powerpoint for the preparation of illustrations

For manuscripts utilizing custom algorithms or software that are central to the research but not yet described in published literature, software must be made available to editors/reviewers. We strongly encourage code deposition in a community repository (e.g. GitHub). See the Nature Research guidelines for submitting code & software for further information.

Data

Policy information about availability of data

All manuscripts must include a data availability statement. This statement should provide the following information, where applicable:

- Accession codes, unique identifiers, or web links for publicly available datasets
- A list of figures that have associated raw data
- A description of any restrictions on data availability

Data (genes) that support the findings of this study have been deposited in DDBJ with the accession codes, LC530191, LC530192, LC530193, LC530194, LC530195, LC530196, LC530197, LC530198, and LC530199. The data that support the findings of this study are available from the corresponding author upon reasonable request.

Field-specific reporting

Please select the one below that is the best fit for your research. If you are not sure, read the appropriate sections before making your selection.

☒ Life sciences ☐ Behavioural & social sciences ☐ Ecological, evolutionary & environmental sciences

For a reference copy of the document with all sections, see nature.com/documents/nr-reporting-summary-flat.pdf

Life sciences study design

All studies must disclose on these points even when the disclosure is negative.

Sample size	Sample size was not relevant to this study. We repeated experiments and ensured results. Data were reproducible.
Data exclusions	No data were excluded from the analyses.
Replication	All determinations were repeated at least two times.
Randomization	We used different species of thraustochytrids to generate knockout or overexpression strains.
Blinding	GC and LC-ESI MS/MS analysis were performed by blinded member of research group.

Reporting for specific materials, systems and methods

We require information from authors about some types of materials, experimental systems and methods used in many studies. Here, indicate whether each material, system or method listed is relevant to your study. If you are not sure if a list item applies to your research, read the appropriate section before selecting a response.

Materials & experimental systems

n/a	Involved in the study	
☒	☐	Antibodies
☒	☐	Eukaryotic cell lines
☒	☐	Palaeontology
☒	☐	Animals and other organisms
☒	☐	Human research participants
☒	☐	Clinical data

Methods

n/a	Involved in the study	
☒	☐	ChIP-seq
☒	☐	Flow cytometry
☒	☐	MRI-based neuroimaging
